# Purification of Protein Glutaminase by Cell Surface Display and Krill Protein Modification via Deamidation

**DOI:** 10.3390/foods15122107

**Published:** 2026-06-11

**Authors:** Jiacheng Zhang, Yu Zhang, Ting Wang, Xu Li, Xiujuan An, Chong Zhang

**Affiliations:** 1 College of Food Science and Technology, Nanjing Agricultural University, Nanjing 210095, China; 2 College of Animal Science and Technology, Nanjing Agricultural University, Nanjing 210095, China

**Keywords:** CL protein glutaminase, deamidation, cell surface display, krill protein isolate, functional properties

## Abstract

In this study, a novel protein glutaminase derived from *Chryseobacterium lactis* CGMCC 33780 (CLPG) was successfully purified via a one-step cell surface display approach, yielding its mature form. Subsequently, the enzymatic properties of CLPG were characterized. It exhibited optimal activity at a pH of 5 and a reaction temperature of 50 °C, and retained over 70% of its activity after a 12 h incubation at 50 °C. The study further investigated the impact of CLPG-mediated deamidation on the structural and functional attributes of krill protein isolate (KPI). A comprehensive analysis was conducted on the deamidation extent, conformational alterations, and microstructural morphology of KPI, employing techniques such as FTIR, CD, DSC, and SEM. After deamidation treatment with CLPG, the foaming and emulsifying properties of KPI were moderately shifted. When the CLPG dosage was 1.0 U/g with a corresponding deamidation degree of 15.18%, the emulsifying property of KPI reached the maximum value of 23%. These property enhancements were possibly primarily attributed to the increased electrostatic repulsion and hydrophobicity induced during the deamidation process. This work not only pioneers a novel method for the expression and purification of protein glutaminase but also applies it to the modification of krill protein, offering fresh insights for the development and application of protein glutaminases.

## 1. Introduction

Protein engineering and the development of functional biomolecules stand as crucial strategies for addressing the pressing challenges in food science, biomedicine, and industrial biotechnology [1,2]. In the pursuit of sustainable and premium-quality protein resources, krill protein, derived as a byproduct of krill oil extraction, has emerged as a promising candidate, offering a unique fusion of nutritional value, functional versatility, and environmental sustainability [3,4]. Krill oil extraction, a well-established industrial process designed to yield valuable omega-3 fatty acids and antioxidants, generates substantial quantities of krill protein-rich residues that are frequently underutilized [5,6]. Typically discarded or repurposed for low-value applications like animal feed, these residues represent an untapped reservoir of high-quality protein, holding immense untapped potential for the development of protein-based products [7].

Krill protein, derived from the byproduct of krill oil extraction, inherits the distinctive merits of natural krill protein. It boasts a comprehensive amino acid profile, encompassing all the essential amino acids required by humans, and its biological value surpasses that of many conventional meat and dairy proteins [8]. The amino acid composition aligns closely with humans’ nutritional requirements, making it an optimal candidate for the development of functional foods. Thogersen et al. reported that krill protein hydrolysate increases postprandial serum EAA and BCAA concentrations in a superior manner to soy protein isolate and thus might represent a promising future protein source in human nutrition [9]. Furthermore, the unique structural attributes of this krill protein byproduct endow it with favorable physicochemical properties, including commendable solubility and emulsifying capacity, which are of paramount importance to its application in food formulations and the creation of bioactive products [10]. Wang et al. successfully enhanced the curcumin-protecting capability of krill protein by means of succinylation modification [11]. However, native krill protein exhibits unsatisfactory solubility, emulsifying capacity and foaming properties under neutral and weakly acidic conditions, accompanied by poor thermal stability. Therefore, targeted modification research is urgently required to fully explore its functional potential and commercial value. Furthermore, its formal application as a food raw material still requires systematic validation, including toxicological evaluation and allergenicity assessment, and relevant official approval must be obtained prior to practical utilization.

Protein glutaminase is an enzyme capable of catalyzing the deamidation of glutamine residues into glutamic acid, playing a pivotal role in protein modification [12]. This enzymatic modification can markedly alter the charge distribution, solubility, and stability of proteins, thereby enhancing their functional attributes. Li et al. employed PG-modified soy protein isolate-stabilized Pickering emulsions as an innovative delivery system [13]. For krill protein, a byproduct of krill oil extraction, protein glutaminase-mediated modification holds immense promise. As reported previously, enzymatic structural rearrangement via deamidation disrupts the intact spatial conformation of crustacean tropomyosin and destroys conserved IgE-binding conformational epitopes, thereby cutting the intrinsic allergenic potential of krill protein [14]. By inducing controlled deamidation, this enzyme can improve protein digestibility, reduce potential allergenicity, and enhance compatibility with other food ingredients [15], which further justifies our selection of krill protein byproduct as the modification substrate in the present work. This approach effectively addresses the functional constraints of the raw protein byproduct, broadening its application scope in high-value sectors [12,16,17].

Cell surface display stands as a cornerstone biotechnological approach, leveraging genetic engineering to tether exogenous proteins onto the cell surface [18]. This technique demonstrates multifaceted and indispensable utility across diverse fields. In the realm of protein functional research, it illuminates the mechanisms of protein–protein interactions and conformational dynamics [19]. In environmental remediation, it empowers engineered cells to efficiently sequester heavy metals or degrade organic pollutants [20,21]. Within the biomedical sphere, it accelerates the development of whole-cell catalysts and groundbreaking vaccines [22,23]. By endowing cells with precisely tailored functional attributes, cell surface display not only propels fundamental research but also fuels the advancement of sustainable biomanufacturing, laying a robust foundation for tackling critical challenges in biotechnology and associated industries [24].

In this study, we employed a newly characterized protein glutaminase derived from *Chryseobacterium lactis* CGMCC 33780. Compared with conventional PG (derived from *Chryseobacterium proteolyticum*, CPPG), CLPG demonstrates superior catalytic activity against krill protein, with its efficiency reaching approximately 1.2 fold greater than that of CPPG, and it was thus utilized to deamidate the protein. The mature PG was obtained through one-step purification via a cell surface display approach. Subsequently, a suite of analytical techniques—including FTIR, CD, DSC, and SEM—was employed to investigate the functional alterations in the deamidated krill protein, focusing on properties such as foaming and emulsifying capacities. This study is the first to report the modification of krill protein isolate by protein glutaminase to improve its functional properties. It provides novel insights into enzymatic deamidation mediated by novel high-efficiency protein glutaminase, and also lays a theoretical foundation for the in-depth development and industrial application of krill protein resources.

## 2. Materials and Methods

### 2.1. Chemicals, Strains and Plasmids

In this study, tryptone and sodium chloride were purchased from Sangon Biotech (Shanghai) Co., Ltd. Yeast extract was obtained from Oxoid Co., Ltd., Basingstoke, UK. Kanamycin sulfate, trypsin, IPTG and imidazole were supplied by Mreda Technology Co., Ltd. (Beijing). Hydrochloric acid, sulfuric acid, phosphate salts and other metal salts were all purchased from Sinopharm Chemical Reagent Co., Ltd., Shanghai, China. The SDS-PAGE gel preparation kit was bought from Hefei White Shark Biotechnology Co., Ltd., Hefei, China. Ni-NTA affinity resin was obtained from Changzhou Smart-Lifesciences Biotechnology Co., Ltd., Changzhou, China. The lipoprotein Outermemberane Protein A (Lpp ompA) gene was obtained from *Escherichia coli* BL21(DE3) [25]. The protein glutaminase gene was sourced from *Chryseobacterium lactis* CGMCC 33780 and was stored in our lab and underwent codon optimization and synthesization by Sangon Biotech (Shanghai) Co., Ltd., Shanghai, China. The genes of LPP-ompA and CLPG were followed by cloning into the pET28a vector. The KOD polymerase used for PCR was purchased from Toyobo Co., Ltd., Osaka, Japan. The ClonExpress Ultra One Step Cloning Kit was purchased from Vazyme Biotech Co., Ltd., Nanjing, China. Primers used for One Step Cloning were designed by SnapGene and synthesized at Sangon Biotech (Shanghai) Co., Ltd., Shanghai, China.

### 2.2. Bioinformatics Analysis of CLPG

AlphaFold3 was used to build the homologous model of CLPG. Protein function prediction was performed based on UniProt (http://www.uniprot.org/) database searches, and secondary structures were analyzed by Espript 3.0 (http://espript.ibcp.fr/ESPript/cgi-bin/ESPript.cgi, accessed on 21 May 2026). The structure of CLPG was analyzed using Discovery Studio Visualizer and PyMOL 3.1.

### 2.3. Expression of Chitinase in E. coli

The recombinant plasmid pET28a-Lpp-ompA-CLPG was transformed into *E. coli* BL21 (DE3) [26]. The transformant strain, designated as BL21 (DE3)-pET28a-Lpp-ompA-CLPG, was cultivated in Luria–Bertani (LB) medium supplemented with 50 μg mL^−1^ kanamycin at 37 °C and 180 rpm for overnight. Then, the culture was transferred to 50 mL of LB medium with 1% (*v*/*v*) inoculum, which also contained 50 μg mL^−1^ kanamycin, and further grown at 37 °C. When the OD_600_ reached 0.6–0.8, 0.2 mM IPTG was added for gene expression at 20 °C and 180 rpm for 24 h. The cultures were collected by centrifuged at 8000× *g* for 5 min. The bacterial pellets were then resuspended in 10 mL of purification buffer (50 mM Tris-HCl, pH 7.4, containing 20 mM imidazole and 500 mM NaCl) and mixed with trypsin solution at a final concentration of 0.3 mg/mL and incubated at 37 °C for 1 h [27]. This was then centrifuged at 8000× *g* for 5 min and the supernatant was collected.

### 2.4. Purification of Chitinase by Ni-NTA Affinity Chromatography

The collected supernatant was further purified using nickel affinity chromatography, and the target proteins were eluted with imidazole gradient solutions. Prior to sample loading, the chromatographic column was rinsed with ultrapure water and balanced with basal purification buffer (50 mM Tris-HCl, pH 7.4, 20 mM imidazole and 500 mM NaCl). Subsequently, the crude CLPG sample flowed through the Ni-NTA resin under gravity to achieve sufficient binding. Unbound impurities were removed by washing buffer (50 mM Tris-HCl, pH 7.4, 50 mM imidazole and 500 mM NaCl), and the target enzyme was finally eluted with high-concentration imidazole eluent.

SDS-PAGE was adopted to verify the purity of harvested enzyme samples. A NanoDrop Lite spectrophotometer (Thermo Fisher Scientific, New York, NY, USA) was used to detect the protein concentration in eluted fractions. Afterwards, the purified enzyme solution was concentrated and buffer-exchanged into 20 mM PBS (pH 7.4) via ultrafiltration (10 kDa), and preserved at −80 °C for subsequent experiments.

### 2.5. Enzymatic Properties Determination

The deamidation activity of CLPG was evaluated following the method described by Zhang et al. [28]. The optimal reaction pH of CLPG was determined in 50 mM Britton–Robinson buffer within the pH range of 2.0–11.0. For pH stability assessment, the enzyme was pre-incubated in the same buffer system at diverse pH levels spanning 2.0 to 11.0 for 2 h [29]. Following incubation, the residual enzymatic activity was determined using a standard enzyme assay protocol. Three independent biological replicates were performed for all experiments (*n* = 3), which is the most widely adopted repetition number in similar enzymatic modification and functional property determination research, ensuring data stability and eliminating accidental errors. Statistical power analysis was conducted combined with variance analysis to evaluate statistical significance.

The temperature-dependent catalytic activity of CLPG was detected at temperatures from 20 °C to 80 °C in 20 mM sodium phosphate buffer (pH 6.5). To explore its thermal stability, the CLPG solution at 1 mg/mL was incubated in the identical buffer under corresponding temperature conditions for 1–12 h. After heat treatment, the remaining enzyme activity was measured following the standard experimental procedure. The negative control was prepared by firstly mixing the enzyme solution with trichloroacetic acid (TCA) to completely inactivate the enzyme, followed by the addition of substrate for incubation reaction. Finally, the mixture was determined for enzyme activity together with other experimental samples.

The impacts of different metal ions on CLPG catalytic performance were explored under standard reaction conditions. A series of metal chlorides such as KCl, MgCl_2_, CaCl_2_, FeCl_3_, CuCl_2_, MnCl_2_, LiCl, NiCl_2_, BaCl_2_ and CoCl_2_ were separately added into the reaction system at a final concentration of 5 mM for activity determination. This experiment clarified the regulatory effects of these metal ions on CLPG enzymatic activity.

The kinetic constants of purified CLPG were characterized via the established enzymatic activity detection method. The reaction system was supplemented with substrate Cbz-Gln-Gly at final concentrations of 1–50 mM together with adequate purified enzyme. All assays were performed in three independent replicates to guarantee experimental credibility. The kinetic parameters including *K*_m_, *V*_max_ and *K*_cat_ were further calculated using the Lineweaver–Burk plotting method.

### 2.6. Degree of Deamidation (DD) and Hydrolysis (DH)

The DD was measured by the phenol–hypochlorite colorimetric method. Briefly, 100 μL sample solution was blended with 500 μL reagent A, 400 μL distilled water, 250 μL reagent B and 500 μL reagent C in sequence. Among them, reagent A contained 40.06 g/L phenol and 0.15 g sodium nitroprusside; reagent B was 49.94 g/L potassium hydroxide solution; and reagent C consisted of 200.04 g/L potassium carbonate and 835 μL sodium hypochlorite. The mixed system was then incubated at 37 °C for 30 min [30]. In the negative control group, 100 μL of sample solution was replaced with equal volume of distilled water. Subsequently, the absorbance value was recorded at 630 nm, and ammonia content was quantified referring to the established standard curve. For total ammonia detection, equal volumes of 2 mol/L sulfuric acid were mixed with 1% (*w*/*v*) KPI solution, and the mixture was heated at 100 °C for 4 h before further measurement. A blank control was set up in parallel, containing only sulfuric acid without protein addition under identical heating conditions. The DD was calculated as follows:

DD (%) = A1/A0 × 100

where A1 is the free ammonia content of KPI after deamidating by CLPG, whilst A0 signifies the total ammonia content of KPI.

The OPA method was utilized to determine the DH of deamidated KPI (DKPI) samples.

### 2.7. Determination of Turbidity and Surface Hydrophobicity

All samples were dissolved in 20 mM pH 6.5 phosphate buffer and fully blended to prepare 1% (*w*/*v*) protein solution. Solution turbidity was evaluated by detecting absorbance at 500 nm [31].

Turbidity = A × ln(10)/L

where A is the absorbance at 500 nm and L was the optical path of the cuvette.

The Bromophenol blue (BPB) was utilized to analyze the surface hydrophobicity of the samples. The 2 mg KPI was diluted to 1% (*w*/*v*) with 200 μL 20 mM PBS (pH 6.5). Subsequently, 40 μL of Bromophenol blue (1 mg mL^−1^) was added, shake for 2 min, 10,000 rpm for 2 min. The surface hydrophobicity was calculated by measuring the absorbance at 595 nm. We used 20 mM phosphate-buffered saline (pH 6.5) instead of KPI solution as a blank sample.

Bromophenol blue binding capacity (μg) = (200 × A0 − A1)/A0

where A0 is the absorbance of the blank sample supernatant, whilst A2 is the absorbance of the sample supernatant.

### 2.8. Foaming Properties and Emulsifying Properties

The foaming properties and emulsifying activities of the samples were assessed using a previously described method with minor modifications [31]. A 1% (*w*/*v*) KPI/DKPI protein solution was placed in a 10 mL centrifuge tube, and its initial volume was recorded as V0. The sample was then homogenized at 15,000 rpm for 2 min using a high-speed homogenizer, after which the foam volume was measured and recorded as V1. After allowing the foam to stand for 30 min, the remaining foam volume was noted as V2. Foaming capacity (FC) and foaming stability (FS) were calculated according to the following formulas:

FC (%) = V1/V0 × 100

FS (%) = V2/V1 × 100

To analyze the emulsifying properties, 5 mL of protein solution was blended with an equal volume (5 mL) of soybean oil and homogenized at 15,000 rpm for 2 min. Emulsion samples (50 μL) were collected from the bottom of the container immediately after homogenization (0 min) and again after 30 min to assess absorbance. Each sample was diluted in 5 mL of 0.1% (*w*/*v*) sodium dodecyl sulfate (SDS) solution, and its absorbance was measured at 500 nm. The emulsion activity index (EAI) and emulsion stability index (ESI) were subsequently calculated using the following equations:

EAI (m^2^/g) = 2 × 2.303 × A0 × N/(φ × L × C ×10,000)

ESI (min) = 30 × A0/(A0 −A30)

A0 is the absorbance values at 0 min, N is the dilution factor, φ is the volume fraction of the oil phase, L is the path length (L = 1 cm), C is the protein solution concentration (g/mL), and A30 is the absorbance values at 30 min.

### 2.9. Particle Size and Zeta Potential

The particle size distribution and zeta potential of native and deamidated KPI were determined using a Malvern Zetasizer Nano S90 instrument (Malvern Instruments, Worcestershire, UK). Samples were prepared into 0.5 mg/mL aqueous solutions and loaded for analysis, with all measurements carried out in triplicate at 25 °C.

### 2.10. Fourier Transform Infrared (FTIR) and Secondary Structure

A precise measurement of the protein sample was conducted, resulting in a weight of 2 mg. This sample was then combined with pre-dried potassium bromide (KBr) pellets at a mass ratio of 1:100 within a mortar, yielding a homogeneous powder. The powder was subsequently pressed into uniform, transparent wafers using a pellet press machine. The KPI/DKPI was analyzed directly by a Fourier Transform Infrared Spectrometer (Nicolet Summit X, Thermo Fisher Scientific, New York, NY, USA). The scanning of all samples was conducted within the wavelength range of 400–4000 cm^−1^.

Circular dichroism spectroscopy with a JASCO J-1500 spectrometer was applied to analyze the secondary structure of original and deamidated KPI. A 0.01% (*w*/*v*) protein suspension was transferred into the quartz cuvette, and scanning was performed within 190–260 nm at a speed of 100 nm/min. Distilled water served as blank control, and all tests were conducted in triplicate.

### 2.11. Intrinsic Fluorescence Spectra and Differential Scanning Calorimetry (DSC)

KPI and deamidated KPI were diluted to 0.1 mg/mL with 10 mM pH 6.5 PBS. Their intrinsic fluorescence spectra were then acquired via a PerkinElmer Ensight multifunctional microplate reader, with excitation set at 290 nm and emission scanned from 300 to 500 nm.

Thermal characteristics were further explored using a Shimadzu DSC-60plus calorimeter. Approximately 2 mg of the accurately weighed sample was sealed in a dedicated crucible, and heated progressively from 40 °C to 180 °C at a constant rate of 5 °C per minute.

### 2.12. Scanning Electron Microscope (SEM)

The surface morphology of KPI/DKPI was observed using a GeminiSEM 360 scanning electron microscope (Zeiss, Oberkochen, Germany). Prior to imaging, the samples were sputter-coated with gold to enhance conductivity. A representative area of interest was selected, and high-resolution images capturing the surface structure and fine morphology of KPI/DKPI were obtained under optimized magnification settings.

## 3. Results

### 3.1. Bioinformatic and Structural Analysis of CLPG

**Figure 1:**
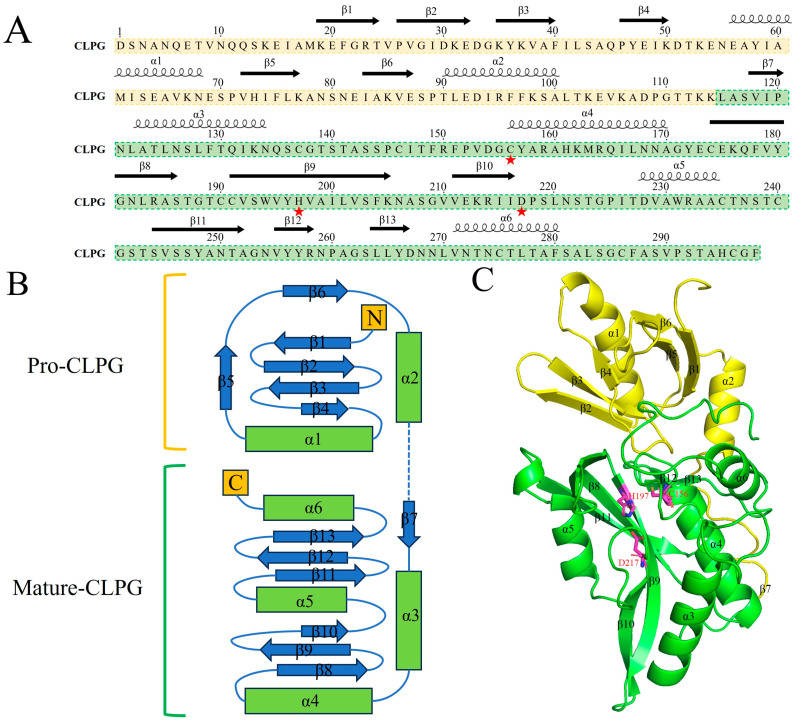
Overall structure of CLPG. (**A**) Protein sequence of CLPG; red stars represent the catalytic triad of CLPG. The yellow shaded part indicates the pro-peptide sequence of CLPG, and the green shaded part indicates the mature peptide sequence of CLPG; (**B**) Overall topology of CLPG. Boxes and arrows represent α-helices and β-sheets, respectively. (**C**) The 3D model of CLPG; the yellow-shaded region represents the pro-peptide, whereas the green-shaded region represents the mature peptide.

A strain capable of producing protein glutaminase activity was screened in the early stage of the laboratory, and it was identified as *Chryseobacterium lactis* after strain identification. Subsequently, the gene encoding the protein glutaminase was obtained through genome sequencing, which contains 298 amino acids. By comparing with other protein glutaminases (Figure S1), this gene contains 1–114 amino acids as the pro-peptide of CLPG and 115–298 amino acids as the mature peptide of CLPG (Figure 1A). Previous studies have shown the catalytic triad of protein glutamine to be CYS-HIS-ASP; therefore, the putative catalytic triad of CLPG as C156-H197-D217 [17]. The three-dimensional structure model of CLPG was predicted using AlphaFold3 (model quality metrics are shown in Figure S2), and its secondary structure and three-dimensional model showed a total of 6 α-helices and 13 β-sheets (Figure 1B,C). The pro-peptide region is composed of two α-helices and six β-sheets, and the mature peptide region is composed of four α-helices and seven β-sheets. The catalytic triad C156-H197-D217 is located between the pro-peptide and mature peptide. Before activation into mature PG, the pro-peptide shields the active pocket, leaving PG in an inactive state [32].

### 3.2. Expression and Purification of CLPG

The LPP and ompA genes used in this study were both derived from BL21 (DE3). The signal peptide of the lipoprotein along with the subsequent nine amino acid sequences was amplified using PCR. Similarly, the gene sequence encoding Asn46 to Asn159 of the Outermembrane Protein A gene was also obtained through PCR. The two genes mentioned above were connected with the CLPG gene and inserted into the pET28a vector through one-step cloning. Since the Lpp and ompA genes are derived from the host itself, no additional sequence optimization is required for their expression.

**Figure 2:**
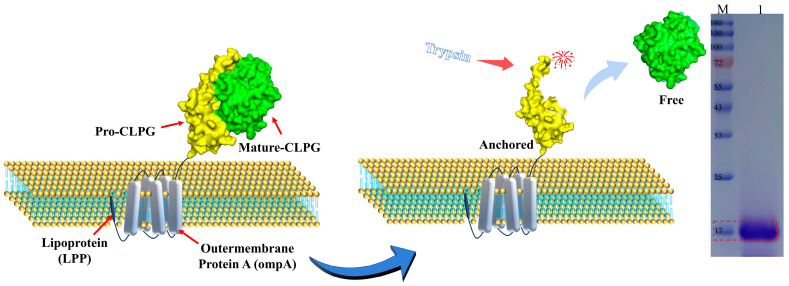
Schematic illustration of Lpp-OmpA-CLPG complex. The pro-peptide region of CLPG is shown in light yellow, and the mature peptide region is shown in green. SDS-PAGE, M: Marker, 1: Mature-CLPG.

Recombinant plasmid pET28a-LPP-ompA-CLPG was constructed and transformed in BL21 (DE3), and the bacterial cells were collected after cultivation and induction. After activation with trypsin and purification, a single band was obtained and verified by SDS-PAGE, with an approximate molecular weight of 17 kDa. The schematic of expression and purification is illustrated in Figure 2. After being fused with a membrane protein, the CLPG protein is transported to the cell membrane surface. At this point, the addition of trypsin cleaves between Pro-CLPG and Mature-CLPG, thereby releasing Mature-CLPG as a free enzyme. Since the C-terminus of Mature-CLPG carries a His-tag, it can be subsequently purified using a Ni-NTA column to obtain highly purified Mature-CLPG.

This method offers certain advantages over the traditional approach of directly expressing CLPG intracellularly. In conventional methods, post-expression cell lysis is required, followed by the purification of CLPG [27]. Subsequently, ultrafiltration must be performed to remove imidazole from the solution, as it could interfere with subsequent experiments. After ultrafiltration, the pro-enzyme activation of CLPG is carried out, and another round of purification is needed. Finally, a second ultrafiltration step is necessary to obtain Mature-CLPG suitable for experimental use. In contrast, the method employed in this study eliminates the need for cell lysis. Instead, only cell collection is required prior to activation. Subsequently, just one round of purification and one ultrafiltration step are sufficient to yield Mature-CLPG ready for experimental application.

### 3.3. Operational and Catalytic Parameters of CLPG

**Figure 3:**
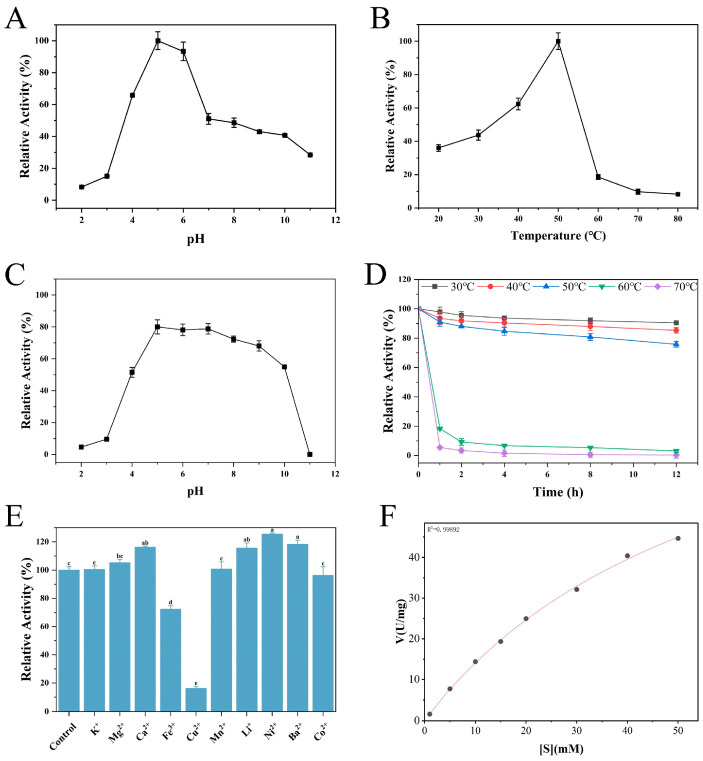
Enzymatic properties of CLPG. (**A**) Optimal reaction pH of CLPG. (**B**) Optimum reaction temperature of CLPG. (**C**) Stability of the CLPG at different pH. (**D**) Thermal stability of CLPG. (**E**) Effect of metal ions on CLPG activity, the statistical significance (*p* < 0.05) is indicated by the use of different letters on each bar. (**F**) Lineweaver–Burk plot of CLPG. Three independent measurements were averaged, and error bars indicate the standard deviation.

The optimal pH and temperature conditions for CLPG are illustrated in Figure 3A,B. The enzyme demonstrates peak activity at a pH of 5.0 and a temperature of 50 °C. It shares the same optimal reaction temperature as the novel protein glutaminase PG5, which was identified by Leng et al. (Table S1) [33]. Within the pH range of four to six, CLPG exhibited a relative activity exceeding 50%; however, when the pH fell below four or rose above six, its relative activity consistently dropped below 50%. As for temperature, although the optimal reaction temperature was 50 °C, at 60 °C, only less than 20% of its activity remained. The results of pH stability are depicted in Figure 3C. CLPG demonstrated relatively stable activity within a pH range of four to ten. Although the specific activity of CLPG was not particularly high at pH values of four and ten, it retained more than 50% residual activity after being exposed to these pH levels for a certain period. The most stable pH value coincided with the optimal reaction pH, under which conditions the residual activity could reach up to 80%. The results of thermal stability are depicted in Figure 3D. In this study, the temperature stability of CLPG was investigated within a range of 30–70 °C over durations spanning from 1 to 12 h. The results indicate that when the reaction temperature was set at 30 °C, CLPG retained approximately 90% of its initial activity after 12 h. At the optimal reaction temperature, the residual activity of CLPG was about 75% following a 12 h incubation. The novel protein glutaminase FBPG, isolated by Long et al., retained merely approximately 40% of its activity after roughly 4 h at its optimal reaction temperature of 40 °C [27]. However, at 60 °C, only 20% of the enzyme’s activity remained after just one hour, and at 70 °C, this dropped to a mere 5% within the same time frame, suggesting near-complete loss of activity. The examination of the impact of metal ions on CLPG activity revealed that Mg^2+^, Ca^2+^, Li^+^, Ni^2+^ and Ba^2+^ showed a slight facilitative effect, while Fe^3+^ and Cu^2+^ inhibited CLPG activity to varying degrees (Figure 3E). Solutions of the substrate Cbz-Gln-Gly were prepared at varying concentrations to determine the reaction kinetic parameters of CLPG. The *K*_m_ and turnover number *K*_cat_ values were calculated using both double-reciprocal plotting and nonlinear regression fitting methods, as illustrated in Figure 3F. The *K*_m_ and *V*_max_ for CLPG were found to be 69.3 mM and 111.5 U/mg, respectively. The *K*_cat_ for CLPG was determined to be 228.2 min^−1^, and the *K*_cat_/*K*_m_ was calculated to be 3.3 min^−1^·mM^−1^.

### 3.4. CLPG-Mediated Modification of KPI

#### 3.4.1. The DD and DH of DKPI

**Figure 4:**
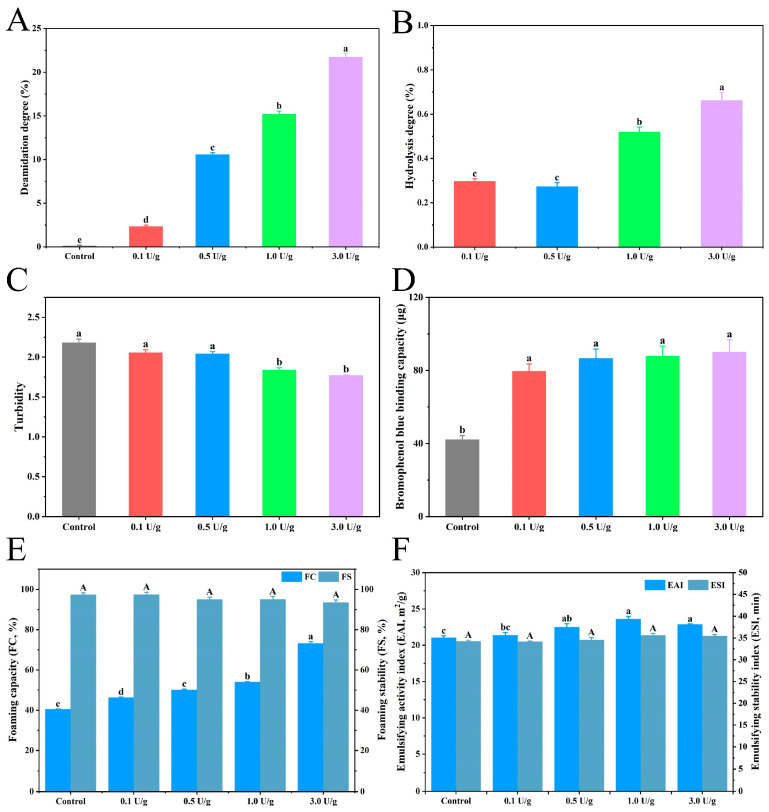
Property changes in KPI and DKPI. (**A**) Deamidation degree. (**B**) Hydrolysis degree. (**C**) Turbidity. (**D**) Surface hydrophobicity. (**E**) Foaming capacity and stability. (**F**) Emulsifying activity and stability. Data are presented as the mean ± s. d. The statistical significance (*p* < 0.05) is indicated by the use of different letters on each bar.

CLPG was employed at varying dosages to catalyze the deamidation of KPI, aiming to enhance its properties. After a one-hour reaction, the extent of deamidation in samples treated with different enzyme-to-substrate ratios (Control, 0.1 U/g, 0.5 U/g, 1.0 U/g, and 3.0 U/g) was evaluated. The corresponding degrees of deamidation were 0.1%, 2.3%, 10.6%, 15.2%, and 21.7%, respectively (Figure 4A; SDS-PAGE is shown in Figure S2), illustrating a dose-dependent increase in deamidation. This further confirmed that the mild thermal conditions employed had negligible impact on amide groups. As illustrated in Figure 4B, the hydrolysis levels across all treatments remained below 0.7%, highlighting CLPG’s high specificity for glutamine residues and its minimal proteolytic activity.

#### 3.4.2. The Turbidity and Surface Hydrophobicity of DKPI

Furthermore, turbidity measurements (Figure 4C) revealed a notable reduction in protein haze as deamidation increased, with values decreasing from 2.2 to 1.8. This effect is likely due to the incorporation of negatively charged carboxyl groups, which enhance protein–water interactions [34]. Surface hydrophobicity is a crucial structural property of proteins. In the three-dimensional structure of proteins, non-polar amino acid residues are distributed on the molecular surface. These exposed hydrophobic domains influence protein properties through hydrophobic interactions. The surface hydrophobicity analysis of KPI treated with CLPG is shown in Figure 4D. All DKPI samples exhibited higher Bromophenol blue binding capacity than native KPI, with the binding amount increasing from an initial 42.1 μg to a maximum of 90.1 μg, representing a 114% increase. This indicates that deamidation promotes the exposure of hydrophobic domains, thereby enhancing the hydrophobicity of DKPI [35].

#### 3.4.3. Changes in Foaming and Emulsifying Properties

Foaming and emulsifying capabilities are also crucial parameters for proteins, as they determine the practical applicability of these macromolecules. To elucidate the impact of CLPG-mediated deamidation on the foaming properties of KPI, we analyzed foaming capacity and foaming stability, as illustrated in Figure 4E. These parameters are closely related to protein structure, solubility, surface hydrophobicity, among other factors. The results demonstrate that after CLPG-mediated deamidation, the foaming ability of KPI was significantly enhanced, increasing from 40.4% for native KPI to 73.1%. This improvement aligns with our earlier finding that deamidation increases the surface hydrophobicity of KPI. Protein surface hydrophobicity is a key driving force for migration toward the air–liquid interface; thus, the increased hydrophobicity following deamidation accelerates the adsorption of KPI at the interface, thereby enhancing foam formation. However, foaming stability remained largely unchanged, likely because the integrity of protein–protein interactions (such as hydrogen bonds and disulfide bridges) was preserved, maintaining the strength of the interfacial film. Consequently, while foam generation improved, the stability of the resulting foam did not show significant alteration [36].

The emulsifying performance of KPI showed a slight improvement, increasing from 21% to a maximum of 23%, and this minor 2% increment should not be overemphasized as a prominent functional promotion. From the perspective of surface chemistry, moderate negative charges generated by CLPG-mediated deamidation promote polypeptide unfolding and facilitate protein adsorption onto oil droplet surfaces, which is the core reason for the mild rise in EAI. The emulsifying capacity reflects the ability of proteins to form new emulsion interfaces, and its enhancement may be attributed to increased protein surface hydrophobicity and improved solubility. However, emulsion stability remained largely unchanged, mainly because excessive negative charges on deamidated KPI hinder tight molecular packing at the oil–water interfacial layer. Films stabilized solely by hydrophobic interactions are relatively fragile and unable to effectively prevent oil droplet coalescence.

### 3.5. CLPG-Regulated Functional Properties of KPI

#### 3.5.1. Particle Size and Zeta Potential

**Figure 5:**
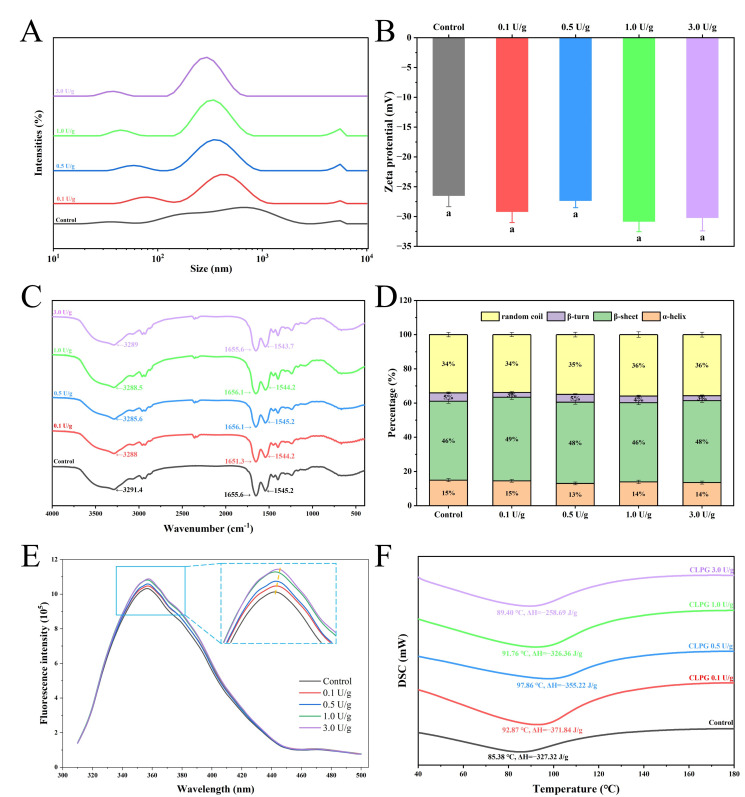
Effects of CLPG-mediated deamidation on protein properties of KPI and DKPI. (**A**) Size distribution. (**B**) Zeta potential, the statistical significance (*p* < 0.05) is indicated by the use of different letters on each bar. (**C**) FTIR spectra. (**D**) Secondary structure. (**E**) Fluorescence spectra. (**F**) DSC thermograms.

To investigate the impact of CLPG-mediated deamidation on KPI, we also analyzed the particle size distribution (Figure 5A) and zeta potential (Figure 5B) of deamidated KPI. As shown in Figure 5A, native KPI exhibited a broad particle size distribution. With increasing CLPG enzyme concentration and progressive deamidation, the particle size gradually decreased. The deamidation reaction, which converts glutamine to glutamic acid, may disrupt existing hydrogen bonding networks and non-covalent interactions, promoting subunit dissociation or the loosening of the protein structure [37]. Furthermore, as glutamine is converted to glutamic acid, the number of negative charges on the protein surface increases significantly, generating electrostatic repulsion that prevents further particle aggregation. This observation is supported by the zeta potential results: with progressive deamidation, the theoretical increase in negative surface charges enhances electrostatic repulsion, thereby increasing the absolute value of the zeta potential [38].

#### 3.5.2. FTIR Spectra and Secondary Structure

To further elucidate the mechanism of CLPG-mediated deamidation, structural changes in KPI were analyzed using Fourier-transform infrared spectroscopy (FTIR) and circular dichroism (CD) spectroscopy. As shown in Figure 5C, the FTIR spectra of all deamidated KPI samples closely resembled that of native KPI, confirming that CLPG-mediated deamidation does not disrupt the protein’s backbone structure. Key characteristic absorption bands included those corresponding to N–H/O–H stretching vibrations (3300–3400 cm^−1^), Amide I band (1600–1700 cm^−1^), and Amide II band (1530–1550 cm^−1^). After deamidation, a red shift was observed in the N–H/O–H peak, likely due to the generation of additional polar groups, which enhance dipole moment interactions and thereby induce this spectral shift. The Amide I band initially exhibited a red shift followed by a blue shift during deamidation. This may be attributed to carboxylic acid formation, which engages in hydrogen bonding with surrounding water molecules or polar groups, reducing the force constant of C=O bonds and lowering their vibrational frequency. However, as the reaction progresses, pre-existing hydrogen bonds may break due to steric hindrance or charge repulsion, restoring higher vibrational frequencies for C=O bonds. In contrast, minimal changes were observed in the Amide II band, suggesting that this region is relatively insensitive to deamidation.

Figure 5D illustrates the changes in secondary structure of KPI following deamidation. Overall, as the degree of deamidation increased, α-helix content slightly decreased. The stability of α-helices relies on intrachain hydrogen bonds; however, carboxylic acids generated during deamidation carry negative charges that may disrupt pre-existing hydrogen bonding through electrostatic repulsion, leading to loosening of helical structures. This, in turn, promotes increases in β-sheet and random coil contents. These findings indicate that deamidation alters protein charge distribution and intermolecular forces, ultimately resulting in targeted remodeling of secondary structure [39].

#### 3.5.3. Changes in Fluorescence Spectra and Thermal Stability of KPI

Intrinsic fluorescence analysis (Figure 5E) was utilized to monitor the exposure of tryptophan residues. The maximum emission wavelength (λmax) serves as a sensitive indicator of changes in the polarity of the microenvironment surrounding hydrophobic residues. The results revealed a consistent red shift in the intrinsic fluorescence spectra of deamidated KPI, indicating partial unfolding of the protein structure and increased exposure of hydrophobic amino acid residues [40].

The thermal stability of proteins is closely related to their spatial conformation. As shown in Figure 5F, the denaturation temperature of KPI initially increased from 85.38 °C to a peak of 97.86 °C following CLPG-catalyzed deamidation, but subsequently decreased with further increases in deamidation extent, ultimately dropping back to 89.4 °C. At a low CLPG dosage, mild deamidation introduces a moderate amount of negative charges on polypeptide chains, eliminating steric hindrance among protein molecules and facilitating ordered intermolecular rearrangement to form stable, compact β-sheet networks. The elevated proportion of regular β-sheet consequently strengthens structural rigidity and raises the denaturation temperature of KPI [41]. In contrast, excessive deamidation at high enzyme loading accumulates abundant carboxylate anions and excessive negative surface charges, triggering intense intramolecular and intermolecular electrostatic repulsion. Although β-sheet content continues to rise at 3.0 U/g, the newly formed β-sheet domains are under severe structural strain and evolve into an unstable molten globule-like conformation instead of rigid ordered aggregates. However, extensive deamidation leads to significant accumulation of carboxylate anions, generating strong electrostatic repulsion that disrupts hydrophobic interactions. Consequently, the protein loses its ability to maintain a compact conformation, leading to reduced α-helix content and increased random coil formation. These strained, unstable β-sheet assemblies require less thermal energy for unfolding, thereby inducing a remarkable drop in denaturation temperature at high deamidation degrees, which is well consistent with the changing trend of secondary structure determined by CD spectroscopy.

#### 3.5.4. Surface Morphology of Deamidation-Regulated KPI

**Figure 6:**
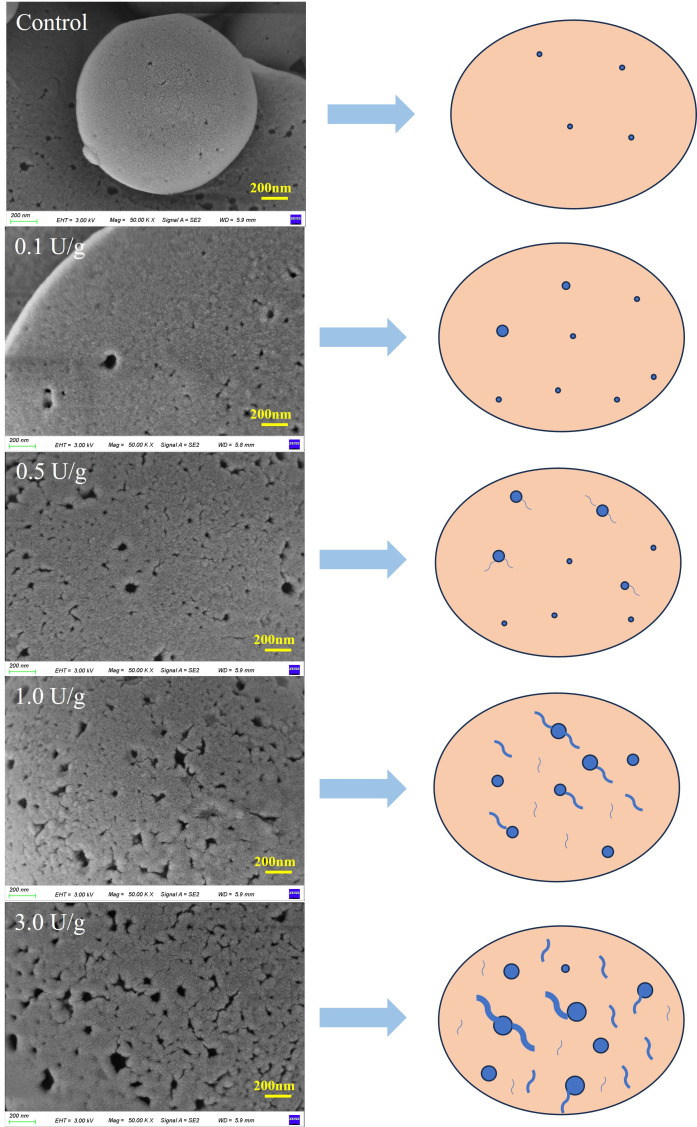
SEM micrographs of KPI and DKPI at varying degrees of deamidation; scale bar = 200 nm. Schematic surface of deamidated KPI on the right.

Scanning electron microscopy (SEM) was employed to examine the surface morphological changes in KPI induced by deamidation (Figure 6). The control group exhibited a relatively smooth protein surface with only a few small pores. However, as the degree of deamidation increased, the SEM image of the 0.5 U/g group revealed not only an increase in surface pores but also the appearance of fine cracks, which were absent in earlier groups. With further increases in deamidation extent, the pores became larger and more numerous, while the cracks widened and increased in number. These observations suggest a transition from protein aggregation to dispersion, likely driven by enhanced electrostatic repulsion between proteins as deamidation progresses; this phenomenon is consistent with our previous experimental findings.

## 4. Discussion

Nevertheless, this study mainly focuses on enzyme preparation and preliminary modification effects, with certain limitations remaining. The structural and functional variations in modified krill protein were analyzed based on experimental phenomena and literature, while quantitative structure–function correlation and in-depth mechanistic exploration were not covered in the current research scope. In addition, key indicators including rheological properties, digestibility and bioavailability of modified protein were not investigated, and the application potential of the enzyme was only verified in pure laboratory systems, lacking validation in real food matrices.

To address the above limitations, we will carry out targeted follow-up research. Future work will focus on establishing the quantitative correlation between structural changes and functional improvements of modified krill protein. Rheological analysis and digestibility evaluation will be supplemented to enrich the basic property data of modified products. Moreover, practical validation in real food matrices will be performed to explore the industrial application potential of this enzymatic modification strategy. Additionally, molecular simulation will be utilized to clarify the molecular mechanism of enzyme-mediated krill protein modification, further improving the theoretical system of this research field.

## 5. Conclusions

This study demonstrates that the novel protein glutaminase CLPG, derived from *Chryseobacterium lactis* CGMCC 33780, can be purified via a one-step cell surface display approach. In contrast to traditional *E. coli*-based expression protocols for protein glutaminase, our approach eliminates a single purification and ultrafiltration step and greatly reduces experimental time consumption. Moreover, its deamidation of krill protein effectively modulates the protein’s structural and functional properties. Specifically, the deamidated Antarctic krill protein exhibits enhanced solubility, foaming capacity (up by 80.9%), and emulsifying capability. These improvements are primarily attributed to the proper exposure of hydrophobic domains and the alteration of electrostatic interactions following deamidation. These findings were further corroborated through analyses using FTIR, CD, and DSC.

This work not only presents a novel approach for the expression and purification of protein glutaminase but also underscores enzymatic deamidation as a highly effective protein modification strategy. The enhanced properties of deamidated krill protein following this treatment significantly elevate its practical application value. While this study preliminarily demonstrates that CLPG-catalyzed deamidation can structurally and functionally modify proteins, the precise deamidation sites and the underlying interaction mechanisms between deamidated protein subunits remain unresolved. Future research will therefore focus on elucidating these mechanisms.

## Data Availability

The original contributions presented in this study are included in the article/Supplementary Materials. Further inquiries can be directed to the corresponding authors.

## Supplementary Materials

The following supporting information can be downloaded at: https://www.mdpi.com/article/10.3390/foods15122107/s1, Table S1. Comparison of Properties of Protein Glutaminases from Different Sources. Table S2. Statistical power and effect size analysis of experimental indexes. Table S3. Purification summary table. Figure S1. Activities of CPPG and CLPG toward krill protein. Figure S2. sequence comparison. Figure S3. Model-quality metrics. Figure S4. Lineweaver-Burk plot. Figure S5. SDS-PAGE Analysis of KPI after Deamidation. Reference [42] is cited in Supplementary Materials.
