# Supplementary material for: Purification of Protein Glutaminase by Cell Surface Display and Krill Protein Modification via Deamidation
Source: Foods. 2026 Jun 11;15(12):2107. doi: 10.3390/foods15122107 (PMC13298967; doi:10.3390/foods15122107)
Supplement: Supplementary file 1 [file foods-15-02107-s001.zip › foods-4296016-supplementary.pdf]

Supporting information

Purification of Protein Glutaminase by Cell Surface Display and Krill Protein Modification via Deamidation

Table S1. Comparison of Properties of Protein Glutaminases from Different Sources 2
Table S2. Statistical power and effect size analysis of experimental indexes 3
Table S3. Purification summary table 4
Figure S1 Activities of CPPG and CLPG toward krill protein 5
Figure S2 sequence comparison 6
Figure S3 Model-quality metrics 7
Figure S4 Lineweaver-Burk plot 8
Figure S5 SDS-PAGE Analysis of KPI after Deamidation 9

Table S1. Comparison of Properties of Protein Glutaminases from Different Sources

Table S1. Comparison of Protein Glutaminase with Other Reported Enzymes

Source	Expressing host	Mol.wt (kDa)	Optimum pH	Optimum temperature	Activity	Reference
Chryseobacterium proteolyticum 9670T	*Chryseobacterium proteolyticum* 9670T	19.8	6.5	60 ℃	1.58 U/mL	1
Chryseobacterium sediminis CGMCC 33779	*Chryseobacterium sediminis* CGMCC 33779	17	5	60 ℃	2.56 U/mL	2
Chitinophaga sp	*E.coil* BL21 (DE3)	17	7	50 ℃	10.04 U/mg	3
Flavobacterium sp. 316	*E.coil* BL21 (DE3)	20.13	6	40 ℃	1.43 U/mg	4
Chryseobacterium lactis CGMCC 33780	*E.coil* BL21 (DE3)	17	5	50 ℃	24.9 U/mg	This study

Table S2. Statistical power and effect size analysis of experimental indexes

	Type I error α	Cohen’s d	Post-hoc power
Effect of metal ions on CLPG activity	0.05	3.448	0.99>0.8
Deamidation degree	0.05	3.120	0.99>0.8
Hydrolysis degree	0.05	0.215	0.12＜0.80
Turbidity	0.05	1.684	0.99>0.8
Surface hydrophobicity	0.05	16.370	0.99>0.8
Foaming capacity	0.05	3.084	0.99>0.8
Foaming stability	0.05	0.275	0.17＜0.8
Emulsifying activity	0.05	3.257	0.99>0.8
Emulsifying stability	0.05	1.929	0.99>0.8
Zeta potential	0.05	2.504	0.99>0.8

Table S3. Purification summary table

	Volume (mL)	Totol protein (mg)	Totol enzyme activity (U)	Specific activity (U/mg)	Purification fold	Yield (%)
Supernatant from enzyme digestion	10	11.2	70.8	6.3	1	100
Ni-NTA	8	2	46.7	23.4	3.7	66
Ultrafiltration	0.6	1.8	43	23.9	3.8	61

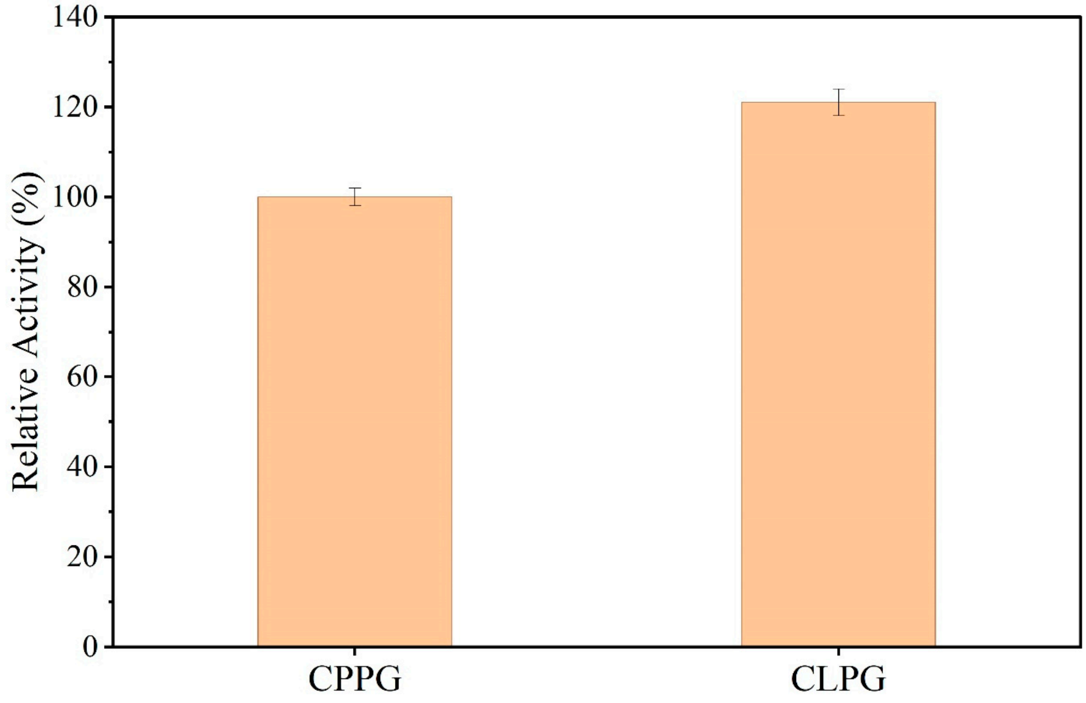

Figure S1 Activities of CPPG and CLPG toward krill protein

```
                         1          10          20          30
>CLPG  .................DSNANQETVNQQ...SKEIAMKEFGRTVPVGIDKEDGK.YKVA
>CSPG  ...............DSGANQDPNLVAKE...SNEIAMKDFGKTVPVGIEKEDGK.FKVS
>CPPG  .................DSNGNQEINGKEKLSVNDSKLKDFGKTVPVGIDEENGM.IKVS
>PG5   MKKLFLGFIAVLALTVVSIVSCKKSEQTPISTPADNDIVLLGNYIPFSYNVNGKEDATVG
>FBPG  ...MKKNLLKLTLLLGLAIVLGSCNNEQEKNPQEVNPQTSNDYIIGLIKKCDDCEVSTSK

       40        50          60        70        80         90
>CLPG  FILSAQPYEIKDTKENE.AYIAMISEAVKNESPVHIFLKANSNE..IAKVESPTLED...
>CSPG  FMVSAQPYQIKDTKENA.GFISMIKEAVENETPVQIFLKANTSE..IAKVDKATAED...
>CPPG  FMLTAQFYEIKPTKENE.QYIGMLRQAVKNESPVHIFLKPNSNE..IGKVESASPED...
>PG5   FLQSAQPFSVDPSKAANGAYVDLLKAGIDKSTPVEVYVYRNTRT..IAKVKPASDEA...
>FBPG  TFFYCGIKAGASYVENDSPYLNDIKESISNGTPIKIYFDENEPERIISVVKISNSEEKNW

             100       110       120       130       140       150
>CLPG  ...IRFFKSALTKEVKADPGTTKKLASVIPNLATLNSLFTQIKNQSCGTSTASSPCITFR
>CSPG  ...IRYFKSILTKEVKEES...RKAVSVIPNLATLNSLFAQIKNQACGTSTASSPCITFR
>CPPG  ...VRYFKTILTKEVKGQTN...KLASVIPDVATLNSLFNQIKNQSCGTSTASSPCITFR
>PG5   ...MARYRQALVAPAKTEALP......TIPSEAALTTLFNQLK.........AAPIPFK
>FBPG  NLNVKYDETPFKTIEDLNRKVSTQKASSFDFTTTEAVNFFNAMKNKSCAINNQNLCIPFQ

             160       170       180          190       200
>CLPG  FPVDGCYARAHKMRQILNNAGYECEKQFVYGNLRA...STGTCCVSWVYHVAILVSFKNA
>CSPG  YPVDGCYARAHKMRQILLNAGYDCEKQFVYGNLRA...STGTCCVSWVYHVAILVSFKNA
>CPPG  YPVDGCYARAHKMRQILMNNGYDCEKQFVYGNLKA...STGTCCVAWSYHVAILVSYKNA
>PG5   FASDGCYARAHKMRQMILAAGYDADKLFVYGNLAA...STGTCCVSWSYHVAPLVNVKTA
>FBPG  YANDGCYARAHMMRQHMNYASKDCYKIFAYGNLKVNTSSTGVCGIAWRYHVAPLISVN..

       210       220       230       240        250       260
>CLPG  SGVVEKRIIDPSLNSTGPITDVAWRAACTNSTCGSTS.VSSYANTAGNVYYRNPAG.SLL
>CSPG  SGIVEKRIIDPSLFSSGPVTDAAWRAACTNTSCGSAS.VSSYANTAGNVYYRSPAG.SLL
>CPPG  SGVTEKRIIDPSLFSSGPVTDTAWRNACVNTSCGSAS.VSSYANTAGNVYYRSPSN.SYL
>PG5   NGTVQQRILDPSLFT.APVAVSTWLNACRNTGCVSTANYTTTRQMPGAVYFIASTGNSPL
>FBPG  ...GVWNVIDPSLFN.QPVTITTWLNKMKYNGG....TVATTSYQNSSVYYYDYVSNYTQ

           270       280        290
>CLPG  YDNNLVNTNCTLTAFSALSGCFAS.VPSTAHCGF
>CSPG  YDNNYVNTNCVLNIFSSLSGCSPSPAPSVGSCGF
>CPPG  YDNNLINTNCVLTKFSLLSGCSPSPAPDVSSCGF
>PG5   YDNSYAHTNCVIAGYTGLVGCGIPPTLNCPL...
>FBPG  YDNNYTDTYSTLANYRYRQTSCSF..........
```

Figure S2 sequence comparison

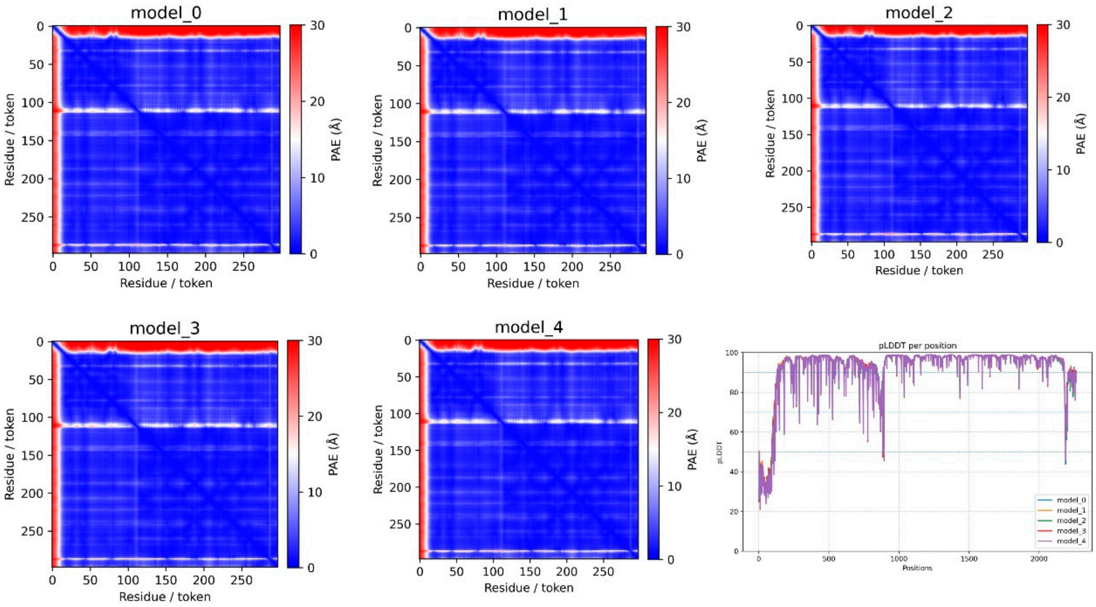

Figure S3 Model-quality metrics

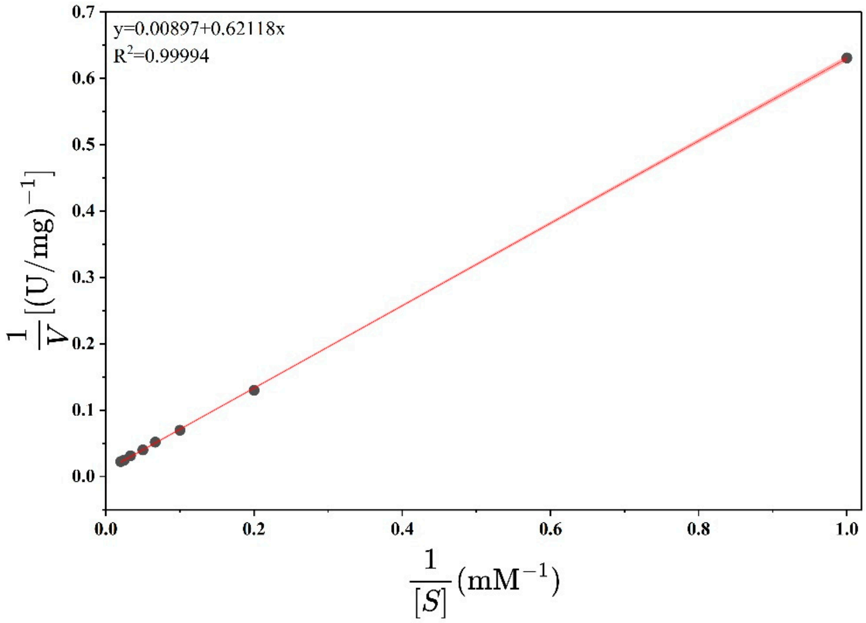

Figure S4 Lineweaver-Burk plot

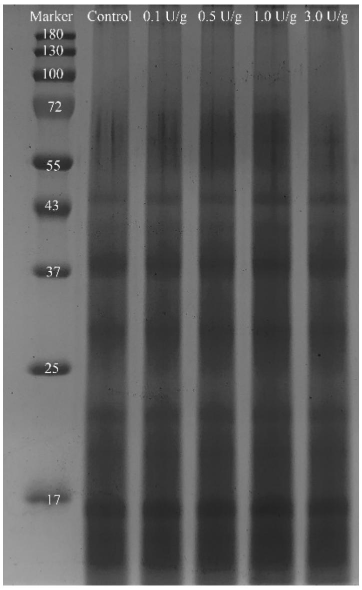

Figure S5 SDS-PAGE Analysis of KPI after Deamidation

Reference

(1) Zhang, K.; Lyu, Y.; Zhang, L.; Zhang, C. Enhanced protein-glutaminase production from Chryseobacterium proteolyticum by the addition of leucine. *Process Biochem* **2023**, *130*, 401-408. DOI: 10.1016/j.procbio.2023.05.001.

(2) Xu, Y. L.; Zhang, Y.; Ma, J. C.; Wang, T.; Zhang, C. Curcumin encapsulation enhanced by soy protein isolate modified through deamidation and pH-shifting: Mechanistic insights using a novel protein-glutaminase. *Food Chem* **2026**, *500*. DOI: ARTN 147498
10.1016/j.foodchem.2025.147498.

(3) Leng, W. J.; Li, Y.; Liang, X.; Li, X. T.; Gao, R. C. Discovery and mechanistic analysis of a novel source protein glutaminase PG5 and its potential application. *Food Chem* **2024**, *457*. DOI: ARTN 140121
10.1016/j.foodchem.2024.140121.

(4) Long, Y. T.; Peng, S. D.; Zhou, Y.; Zhang, H. F.; Zhao, G.; Wang, Y. H. Structural Analysis of Marine Protein Glutaminase Reveals a "Gatekeeper" Residue Affecting Its Catalytic Activity. *J Agr Food Chem* **2024**, *72* (49), 27504-27512. DOI: 10.1021/acs.jafc.4c04471.
